# Dual Targeting Biomimetic Liposomes for Paclitaxel/DNA Combination Cancer Treatment

**DOI:** 10.3390/ijms150915287

**Published:** 2014-08-29

**Authors:** Guo-Xia Liu, Gui-Qing Fang, Wei Xu

**Affiliations:** 1 Department of Clinical Lab, Jinan Stomatological Hospital, Jinan 250001, China; E-Mails: runrun20070606@sina.com (G.-X.L.); fangguiqing123@126.com (G.-Q.F.); 2 Department of Pharmacy, Shandong Provincial Qian Foshan Hospital, Jinan 250014, China

**Keywords:** hyaluronic acid, folate, biomimetic liposomes, paclitaxel, DNA, co-delivery

## Abstract

Combinations of chemotherapeutic drugs with nucleic acid has shown great promise in cancer therapy. In the present study, paclitaxel (PTX) and DNA were co-loaded in the hyaluronic acid (HA) and folate (FA)-modified liposomes (HA/FA/PPD), to obtain the dual targeting biomimetic nanovector. The prepared HA/FA/PPD exhibited nanosized structure and narrow size distributions (247.4 ± 4.2 nm) with appropriate negative charge of −25.40 ± 2.7 mV. HA/FA/PD (PTX free HA/FA/PPD) showed almost no toxicity on murine malignant melanoma cell line (B16) and human hepatocellular carcinoma cell line (HepG2) (higher than 80% cell viability), demonstrating the safety of the blank nanovector. In comparison with the FA-modified PTX/DNA co-loaded liposomes (FA/PPD), HA/FA/PPD showed significant superiority in protecting the nanoparticles from aggregation in the presence of plasma and degradation by DNase I. Moreover, HA/FA/PPD could also significantly improve the transfection efficiency and cellular internalization rates on B16 cells comparing to that of FA/PPD (*p* < 0.05) and PPD (*p* < 0.01), demonstrating the great advantages of dual targeting properties. Furthermore, fluorescence microscope and flow cytometry results showed that PTX and DNA could be effectively co-delivered into the same tumor cell via HA/FA/PPD, contributing to PTX/DNA combination cancer treatment. In conclusion, the obtained HA/FA/PPD in the study could effectively target tumor cells, enhance transfection efficiency and subsequently achieve the co-delivery of PTX and DNA, displaying great potential for optimal combination therapy.

## 1. Introduction

Cancer is the leading cause of deaths worldwide, and thus a seriously threat to human health. Due to the complexity of tumorigenesis and difficulty of cancer therapy, combinations of chemotherapeutic drugs with other treatment modalities like nucleic acid have shown great promise in cancer therapy [1,2]. Paclitaxel (PTX) was an effective chemotherapeutic drug and widely used for cancer treatment, including melanoma, ovarian cancer and hepatoma [3,4]. However, more and more cancers have shown decreased response to the mono-chemotherapy of PTX [5,6], thus, it is necessary to combine gene therapy with PTX to achieve synergistic combination therapeutic effect.

**Scheme 1:**
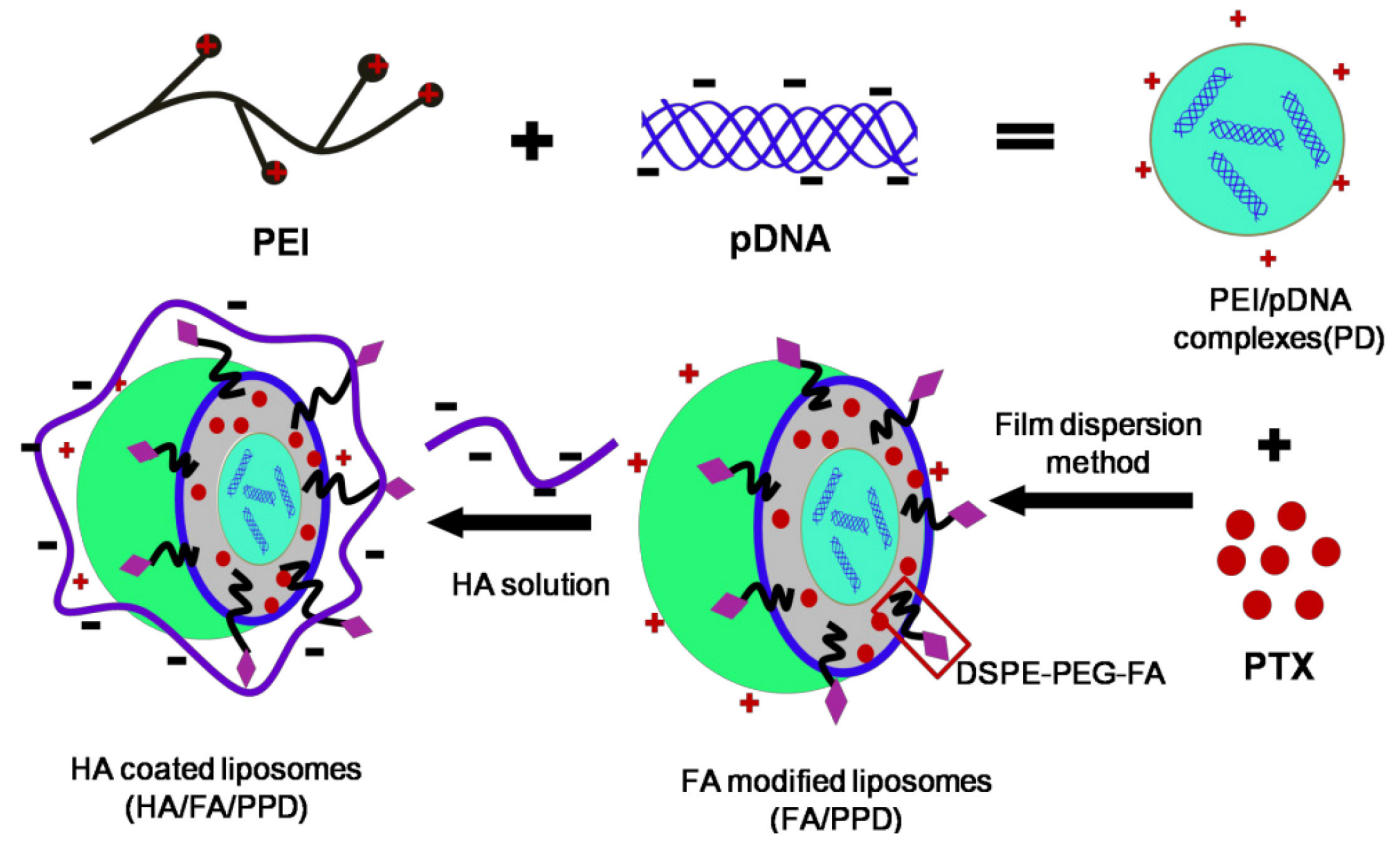
Schematic illustration of formation of HA/FA/PPD (hyaluronic acid (HA) and folate (FA)-modified liposomes). Firstly, PEI (polyethylenimine) bind DNA to form the condensed cationic PEI/DNA complexes, which were chosen as the cationic core of the liposomes. Subsequently, PTX (paclitaxel) and PEI/DNA complexes were co-loaded in the DSPE-PEG_2000_-FA-modified liposomes, forming the FA-modified cationic liposomes (FA/PPD). Lastly, the cationic FA/PPD was added to the anionic HA solution to obtain the HA coated FA/PPD (HA/FA/PPD) by electrostatic attraction.

However, the drug and gene leakage in blood circulation, the non-specific distribution and the immune response to normal tissue seriously limited the clinical application of drug/gene combination treatment [7]. Therefore, it is necessary to design a rational multifunctional nanocarrier to co-deliver PTX and DNA, overcoming the above mentioned problems. Cationic liposomes were reported widely in gene delivery [8,9], and the multilamellar liposomes were expected to show advantages in co-delivering the hydrophobic PTX and the hydrophilic DNA. Considering polyethylenimine (PEI) was one of the most effective nonviral gene delivery polymers [10,11], the condensed PEI/DNA complexes were chosen as the cationic core, to prepare the DNA and PTX co-loaded cationic liposomes (PPD) (as shown in Scheme 1).

In order to achieve efficient drug/gene delivery into target cells, the most promising strategies involved the application of various targeting moieties or ligands that bind specifically to receptors overexpressed on malignant cells [12,13]. Folate (FA), a nontoxic low-weight compound with low immunogenicity, is vital for tumor cell proliferation and survival [14]. The folate receptor (FR) is overexpressed in a wide range of human cancer cells, including ovarian cancer, melanoma, head and neck cancers, presenting an effective means of selectively delivering drug/gene to tumors [15,16]. Therefore, FA was suitable to modify PPD (FA/PPD).

The cationic property of FA/PPD allows it to easily interact with serum complexes compounds, forming aggregates, which are subjected to *in vivo* phagocyte capture, giving rise to vector degradation in turn associated with PTX/DNA release and degradation [17]. Hyaluronic acid (HA), an endogenous component of extracellular matrix, is a negatively charged linear polysaccharide [18,19]. Thus, the negative HA could coat the surface of FA/PPD by electrostatic attraction (HA/FA/PPD), resulting in a biomimetic and anionic layer and subsequently preventing liposomes from aggregating. In *in vivo* circulation, HA coated liposomes could bind specifically to CD44, which is overexpressed in cancer cells [18,19]. In addition, the outer HA layer would inevitably suffer from an enzyme degradation, which would subsequently expose the FA moiety and target to cancer cells again. Consequently, the HA-coated and FA-modified HA/FA/PPD was expected to achieve dual targeting co-delivery of PTX and DNA.

In the present study, the plasmid pCMV-EGFP (pEGFP-N1) carrying enhanced green fluorescent protein (EGFP) were chosen as model gene. HA- and FA-modified dual targeting biomimetic liposomes were prepared by film dispersion method to co-deliver PTX and DNA (HA/FA/PPD), expecting to target to tumor sites, enhance transfection efficiency and realize the optimal combination therapy (as shown in Scheme 1). FA/PPD and PPD were simultaneously prepared to serve as controls. The cytotoxicity of HA/FA/PD (PTX-free HA/FA/PPD) and FA/PD (PTX-free FA/PPD) were studied by 3-(4,5-dimethylthiazol-2-yl)-2,5-diphenyltetrazolium bromide (MTT) assay on murine malignant melanoma cell line (B16) and human hepatocellular carcinoma cell line (HepG2), to evaluate the safety of the nanovectors. The release profile of PTX from HA/FA/PPD was also evaluated by dynamic dialysis method. The stability of HA/FA/PPD and FA/PPD in presence of plasma and the ability to protect DNA against DNase I were investigated to testify the protection function of biomimetic HA layer of HA/FA/PPD. The transfection efficiency and the targeted cellular uptake of HA/FA/PPD, FA/PPD and PPD were evaluated on CD44-positive and FR-positive B16 cells by fluorescence microscope and flow cytometry, respectively [20,21], while the co-delivery efficiency of PTX and pDNA into the same tumor cell was evaluated on CD44-positive and FR-negative HepG2 cells [22,23].

## 2. Results and Discussion

### 2.1. Formation of PEI/DNA Complexes

**Figure 1:**
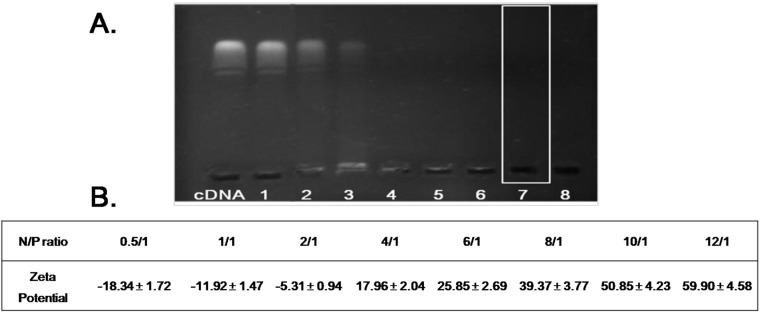
Formation of PEI/DNA complexes. (**A**) Agarose gel electrophoresis retardation assay of PEI/DNA complexes with different N/P ratio. Lanes 1–8: PEI/DNA complexes at N/P ratios of 0.5/1, 1/1, 2/1, 4/1, 6/1, 8/1, 10/1 and 12/1, respectively; (**B**) Zeta potential of PEI/DNA complexes with different N/P ratio.

The condensation of DNA into small particles as a result of interaction with PEI could protect DNA from degradation by DNase and contribute to high transfection efficiency [24]. In the present study, gel retarding analysis was used to optimize the N/P ratio and evaluate the stability of the PEI/DNA complexes. As shown in Figure 1A, in comparison with naked DNA, DNA band was found in Lane 1–3, indicating that when N/P ratios were set as 0.5/1, 1/1, 2/1, respectively, the obtained PEI/DNA complexes were not stable and DNA was released from PEI/DNA complexes. There was no DNA band in Lane 4 and the zeta potential of PEI/DNA complexes reversed from negative to positive (Figure 1B), indicating that PEI could preliminarily condense DNA at N/P ratio of 4/1. Further increasing the N/P ratio to 6/1, 8/1, 10/1 and 12/1 (Lane 5–8) did not allow for the release of DNA from PEI/DNA complexes. To enhance stability and transfection efficiency of the prepared PEI/DNA complexes, the final N/P ratio was set as 10/1.

### 2.2. Formation and Characterization of FA/PPD

**Figure 2:**
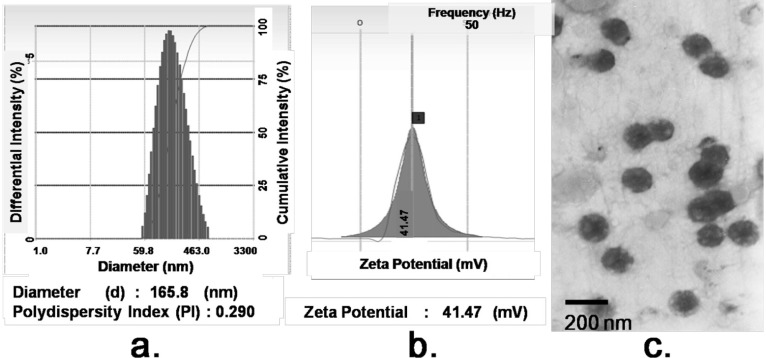
Characteristics of FA/PPD (*n* = 3). (**a**) Particle size; (**b**) Zeta Potential; and (**c**) Transmission electron microscope (TEM) morphology.

FA modified co-loaded PTX and PEI/DNA liposomes (FA/PPD) were successfully prepared via film dispersion method and characterized in terms of morphology, particle size, and surface charge. As illustrated in Figure 2, FA/PPD showed spherical or ellipsoidal shapes with nanosized structure. The average size of FA/PPD was 165.8 ± 4.3 nm and the polydispersity index (PI) was 0.290 ± 0.071, indicating high homogenization of the prepared FA/PPD. Due to the cationic PEI/DNA core, FA/PPD was cationic with positive zeta potential of 41.47 ± 3.24 mV.

### 2.3. Formation and Characterization of HA/FA/PPD

**Figure 3:**
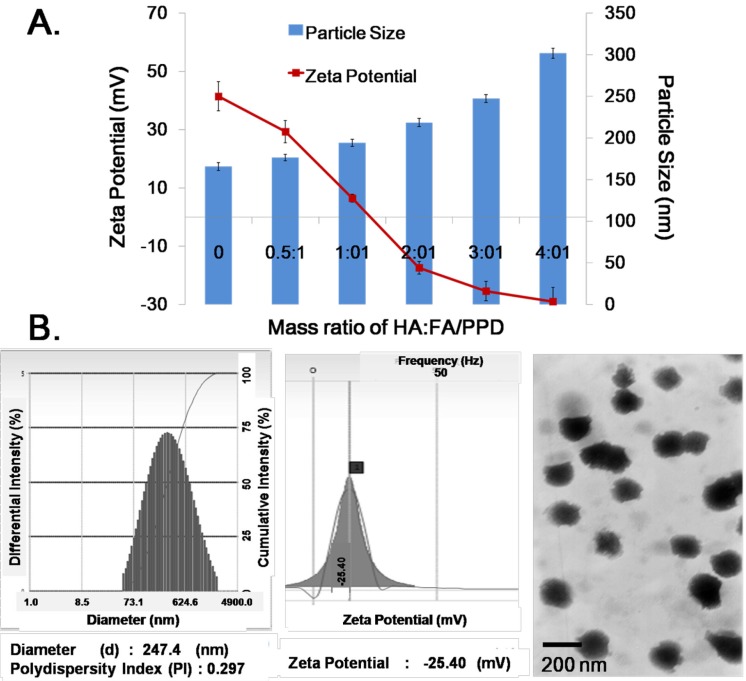
Formation and characterization of HA/FA/PPD (*n* = 3). (**A**) Particle size and zeta potential of liposomes with different mass ratio of HA and FA/PPD; (**B**) Particle size, zeta potential and TEM morphology of finally obtained HA/FA/PPD.

The cationic FA/PPD easily interacted with serum complexes compounds, forming aggregates [17], which are subjected to *in vivo* phagocyte capture, giving rise to vector disintegration in turn associated with DNA release and degradation. To overcome the abovementioned defect, HA, an endogenous component of the extracellular matrix was chosen to protect the cationic FA/PPD. The pKa of HA was approximately 3.0, and the pH of HA solution (300 kDa, 5 mg/mL) in our study was set as pH 6.0. Therefore, the ionization of HA chains was almost complete, and the HA molecule could fully extend. Consequently, HA-coated FA/PPD was prepared by electrostatic attraction between the negative HA and cationic FA/PPD. As shown in Figure 3A, as the assembly process proceeded, the particle size of HA/FA/PPD increased from 172.8 ± 3.6 nm (HA:FA/PPD = 0.5:1) to 291.9 ± 4.2 nm (HA:FA/PPD = 4:1). FA/PPD was coated with a negative layer of HA, therefore, the zeta potential was gradually decreased. When the mass ratio of HA and FA/PPD was 2:1, the zeta potential of HA/FA/PPD reversed from positive to negative charge (−16.59 ± 3.2 mV). In order to keep the nanosized structure of HA/FA/PPD and prevent from aggregating, the optimal mass ratio of HA and FA/PPD was set as 3:1.

After deposition of HA, the HA/FA/PPD was finally obtained, as shown in Figure 3B. The prepared HA/FA/PPD exhibited nanosized structure and narrow size distributions (247.4 ± 4.2 nm, PI = 0.297) with appropriate negative charge of −25.40 ± 2.7 mV. In addition, an obvious HA layered structure was found on the surface of the FA/PPD in the TEM image, demonstrating the successful preparation of HA/FA/PPD.

### 2.4. Stability of HA/FA/PPD in Different Media

#### 2.4.1. Stability of HA/FA/PPD in the Presence of Plasma

**Figure 4:**
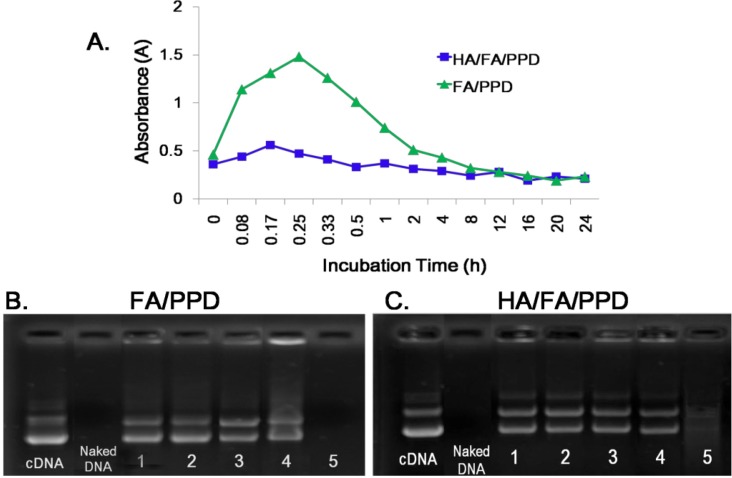
Stability of HA/FA/PPD in different media. (**A**) The turbidity change of HA/FA/PPD in presence of plasma with different incubation time; (**B**) Stability of FA/PPD against DNase I. Lanes 1–5: FA/PPD incubated with DNase I for 0.5, 1, 2, 3 and 4 h, respectively; and (**C**) Stability of of HA/FA/PPD against DNase I. Lanes 1–5: HA/FA/PPD incubated with DNase I for 0.5, 1, 2, 3 and 4 h, respectively.

To estimate the superiority of HA in preventing aggregation of HA/FA/PPD, the turbidity change of HA/FA/PPD suspension in presence of plasma was evaluated and FA/PPD was served as control. The turbidity changes of HA/FA/PPD and FA/PPD after incubation with 50% plasma were shown in Figure 4A. The results showed that FA/PPD with high positive charge aggregated immediately once mixed with the media containing plasma. As incubation time increased, large aggregates began to precipitate, resulting in increased turbidity and absorbance. However, for HA/FA/PPD, the turbidity and absorbance barely changed over time, indicating that HA/FA/PPD was stable and did not aggregate. This phenomenon could be explained that high anionic surface charge of HA prevented serum protein adsorbing onto HA/FA/PPD. Therefore, in comparison with FA/PPD, HA/FA/PPD had higher stability in the presence of plasma and might be more suitable for *in vivo* gene delivery.

#### 2.4.2. Stability of HA/FA/PPD against DNase I

For effective gene expression, DNA in the gene vehicle should be protected from degradation by enzymes [25]. To test whether the prepared HA/FA/PPD and FA/PPD could protect the loaded plasmid DNA from degradation by nucleases, the samples were exposed to DNase I at various time intervals. As illustrated in Figure 4B, naked plasmid DNA was completely digested by DNase I (0.2 U/µg DNA) within 30 min (band intensity of naked DNA was no longer visible). However, DNA in the prepared HA/FA/PPD and FA/PPD still showed a relatively complete structure of supercoiled DNA after incubation with DNase I for 3 and 2 h, respectively. These results showed that HA/FA/PPD and FA/PPD could protect DNA against degradation by DNase I, which is one of the crucial factors for efficient gene delivery *in vitro* as well as *in vivo*.

### 2.5. In Vitro Release of PTX (Paclitaxel) from HA/FA/PPD

**Figure 5:**
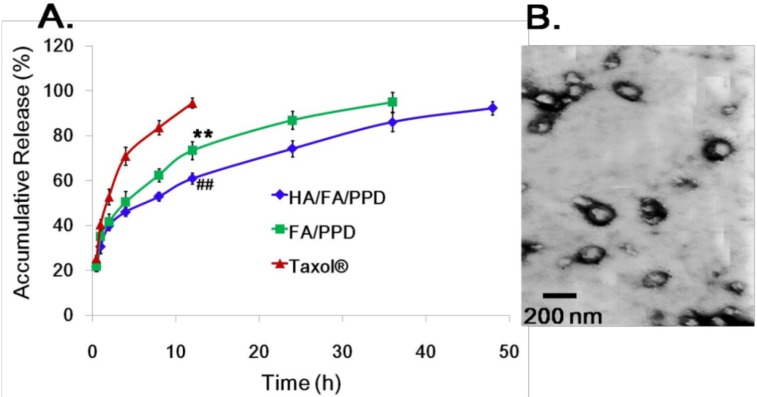
*In vitro* release study. (**A**) *In vitro* release of PTX from HA/FA/PPD, FA/PPD and Taxol^®^; (**B**) TEM morphology of HA/FA/PPDafter 12 h incubation in the release media (*n* = 3). ** *p* < 0.01, statistically significant difference between Taxol^®^ and FA/PPD; ^##^ *p* < 0.01, statistically significant difference between Taxol^®^ and HA/FA/PPD.

To achieve a better antitumor efficiency, the loaded chemotherapy drug PTX should be released completely from the vehicle, therefore, the *in vitro* release study was evaluated by dynamic dialysis method. As shown in Figure 5, within the first 12 h, the release of Taxol^®^ was almost complete (93.15% ± 3.42%), while the release of PTX from HA/FA/PPD (61.09% ± 2.37%) and FA/PPD (72.09% ± 3.02%) was significantly slower than that from Taxol^®^ (*p* < 0.01), avoiding an initial burst drug release and acute toxicity. Moreover, the cumulative amounts of PTX released from HA/FA/PPD and FA/PPD over 36 h were 86.15% ± 2.92% and 94.27% ± 4.01%, respectively, and the remaining PTX in HA/FA/PPD released completely within 48 h (92.26% ± 3.01%). It can be seen from the results that PTX displayed a sustained release phase for HA/FA/PPD and FA/PPD, which probably resulted from the slow erosion and degradation of the nanoparticle components (Figure 5B).

### 2.6. In Vitro Cytotoxicity Studies

**Figure 6:**
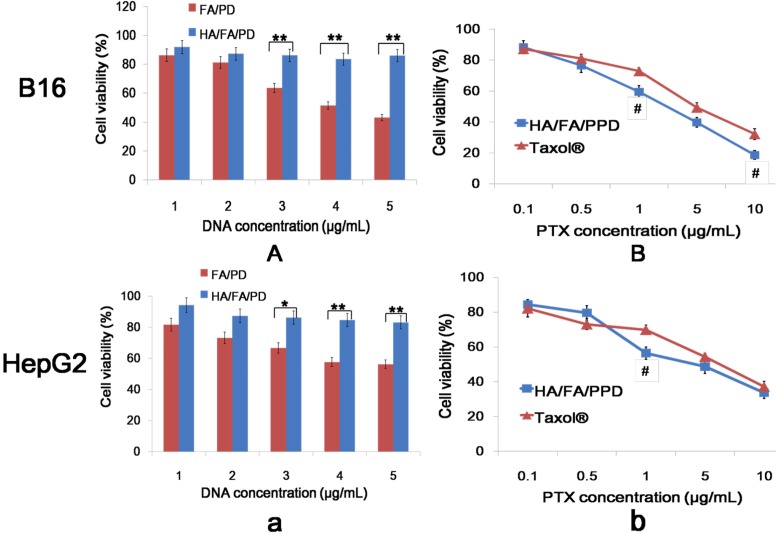
Cytotoxicity of various concentrations of HA/FA/PD, FA/PD, HA/FA/PPD and Taxol^®^ on B16 (**A**,**B**) and HepG2 (**a**,**b**) cells (*n* = 3). * *p* < 0.05, ** *p* < 0.01, statistically significant difference between FA/PPD and HA/FA/PPD; ^#^ *p* < 0.05, statistically significant difference between Taxol^®^ and HA/FA/PPD.

HA/FA/PPD was expected to have low toxicity during transportation in blood and show high toxicity to kill cancer cells in the tumor sites. Therefore, the evaluation of the cytotoxicity of HA/FA/PPD is essential. To evaluate the safety of gene vehicle, HA/FA/PD (PTX free HA/FA/PD) and FA/PD (PTX free FA/PD) were prepared. The *in vitro* cytotoxicity of HA/FA/PD and FA/PD at various DNA concentrations (1.0, 2.0, 3.0, 4.0, and 5.0 µg/mL) was evaluated on B16 and HepG2 cells by MTT assay. As shown in Figure 6A,a, the cell viabilities of B16 and HepG2 cells in presence of HA/FA/PD over the studied concentrations were higher than 80%, significantly higher than that of FA/PD with the same DNA concentrations (*p* < 0.05), demonstrating that the biomimetic HA layer could significantly reduce the cytotoxicity of the nanovehicle.

The *in vitro* cytotoxicity of HA/FA/PPD at various PTX concentrations (0.10, 0.50, 1.0, 5.0, and 10.0 µg/mL) was simultaneously evaluated and Taxol^®^ was served as control (Figure 6B,b). The cytotoxicity of HA/FA/PPD and Taxol^®^, both in a dose-dependent manner and after 48 h incubation, and the IC_50_ values of HA/FA/PPD and Taxol^®^ were calculated. The IC_50_ values of HA/FA/PPD on B16 and HepG2 cells were 1.92 and 3.28 µg/mL, respectively, significantly lower than that of Taxol^®^ on B16 (3.96 µg/mL) and HepG2 cells (5.02 µg/mL). The increased cytotoxicity may be likely attributed to the high delivery efficiency of HA/FA/PPD.

### 2.7. In Vitro Transfection Studies

**Figure 7:**
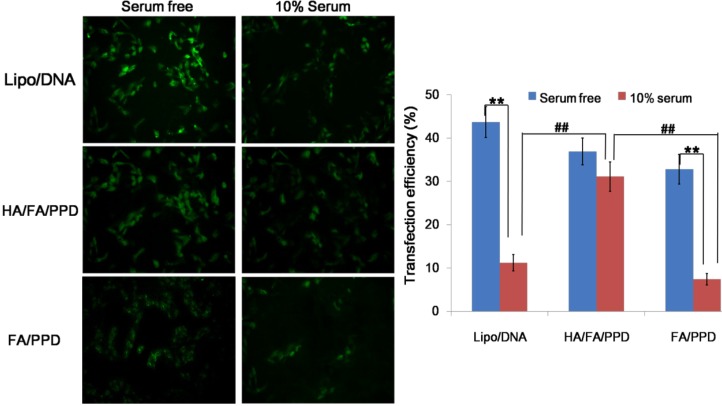
Transfection efficiency of dual targeting HA/FA/PPD on B16 cells (*n* = 3). Lipofectamine™ 2000/pDNA and FA/PPD were served as controls. **Left panel**: Fluorescent micrographs of HA/FA/PPD, FA/PPD and Lipofectamine™ 2000 with or free of 10% serum following 48 h incubation (×20); **Right panel**: Transfection efficiency quantitatively determined by flow cytometry. ** *p* < 0.01, **^##^** *p* < 0.01.

B16 cells were chosen as the both CD44-positive and FA receptor (FR)-positive cell line for *in vitro* transfection studies, and the transfection efficiency of the dual targeting HA/FA/PPD was evaluated by both fluorescence microscope and flow cytometry. As shown in Figure 7, in serum free media, Lipofectamine™ 2000, HA/FA/PPD and FA/PPD could successfully transfer B16 cells in 48 h. Notably, the anionic HA/FA/PPD could obtain high transfection efficiency as well as cationic FA/PPD, which was probably caused by the dual targeting moieties of HA/FA/PPD. However, in the transfection media containing 10% serum, the transfection efficiency of Lipofectamine™ 2000 and FA/PPD was significantly decreased (*p* < 0.01). In comparison with Lipofectamine™ 2000 and FA/PPD, HA/FA/PPD could maintain the nano structure in media containing serum, and display significantly higher transfection efficiency (*p* < 0.01).

### 2.8. In Vitro Targeting Cellular Uptake Studies

**Figure 8:**
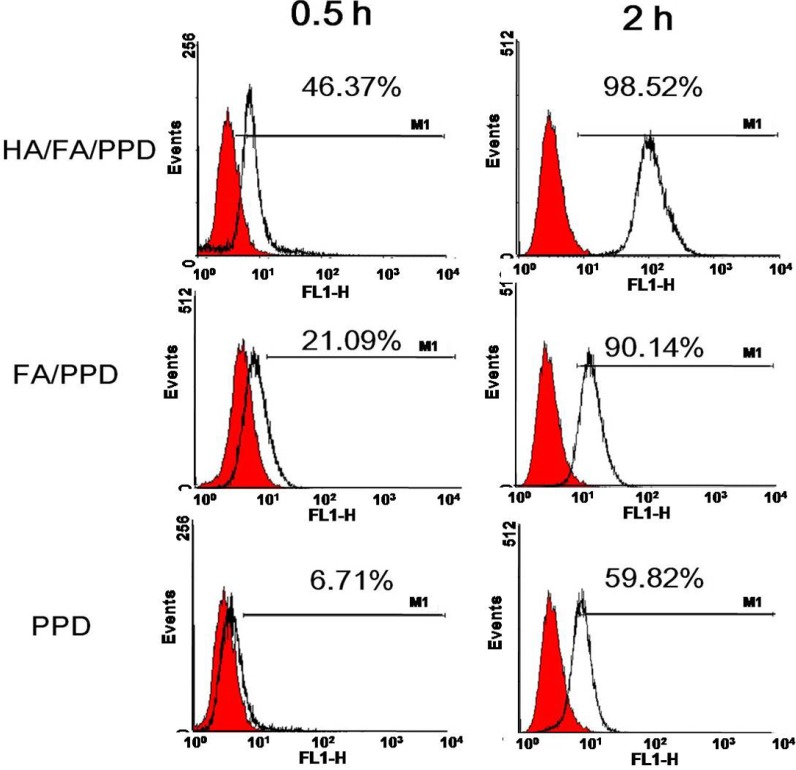
*In vitro* targeting cellular uptake of Rho-PTX (Rhodamine-PTX) labeled HA/FA/PPD on B16 cells (*n* = 3). HA/FA/PPD incubated with B16 cells for 0.5 or 2 h, respectively, while FA/PPD and PPD were served as controls.

To evaluate the dual targeting properties of HA/FA/PPD, the cellular uptake of Rhodamine-PTX (Rho-PTX) labeled (10%, *w*/*w*) HA/FA/PPD was performed on both CD44-positive and FR-positive B16 cells by flow cytometry. As shown in Figure 8, the cellular uptake rate of HA/FA/PPD, FA/PPD and PPD increased over incubation time. Compared to PPD, FA/PPD showed significantly higher internalization rates at 0.5 and 2 h, respectively (*p* < 0.01), which was probably caused by the overexpression of FR on the membrane of B16 cells, resulting in the enhancement of uptake by FR-mediated internalization. Moreover, comparing to FA/PPD, HA/FA/PPD showed significantly higher internalization rates at 0.5 h (*p* < 0.01). Although the internalization rates did not show a significant difference between HA/FA/PPD and FA/PPD at 2 h, the Geometric-mean (G-mean) values showed a great increase (82.42 *vs.* 117.21). These results demonstrated that HA not only served as a protection layer, but also promoted the cellular internalization rates via CD44-mediated internalization. All these results indicated that the dual targeting of HA/FA/PPD could be more likely to reach the target tumor sites and be absorbed by B16 cells, which would lead to the enhancement of therapeutic efficiency.

### 2.9. Co-Delivery of PTX and pDNA to Tumor Cells

**Figure 9:**
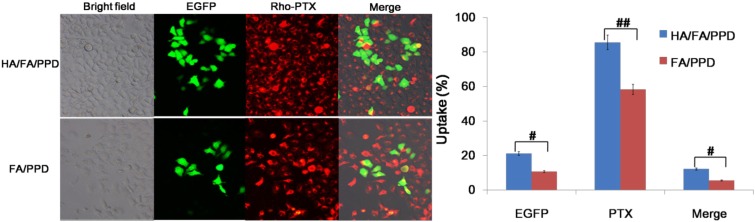
*In vitro* co-delivery efficacy of dual targeting HA/FA/PPD on HepG2 cells (*n* = 3). HA/FA/PPD incubated with HepG2 cells for 4 h, while FA/PPD and PPD served as controls. **^##^** *p* < 0.01, **^#^** *p* < 0.05.

For combined drug and gene cancer treatment, co-delivery of PTX and pDNA into the same tumor cell is important to achieve synergistic therapeutic effect. In present study, the Rho-PTX labeled (10%, *w*/*w*) HA/FA/PPD and FA/PPD was prepared to study the co-delivery efficiency of PTX and pDNA. CD44-positive and FR-negative HepG2 cells were chosen as the model cell lines. As shown in Figure 9, bright green fluorescence was observed, indicating that pDNA was successfully delivered into HepG2 cells and transfected. Meanwhile, red fluorescence of Rho-PTX was also observed in the same cells. The red and green fluorescence could be overlapped in the merged image. These results indicated that the HA/FA/PPD and FA/PPD could co-deliver PTX and pDNA into the same tumor cell, and subsequently show the synergistic antitumor effect, which was promising, to reduce the dose of PTX and enhance therapeutic efficacy. The flow cytometry results confirmed the co-delivery efficiency and further demonstrated that HA/FA/PPD (12.24% ± 1.93%) was superior to FA/PPD in co-delivering PTX and pDNA (5.61% ± 1.14%) into the same tumor cell (*p* < 0.05), indicating that HA could enhance the co-delivery efficiency.

## 3. Experimental Section

### 3.1. Materials

Paclitaxel (PTX), rhodamine labeled paclitaxel (Rhodamine-Paclitaxel, Rho-PTX), cholesterol (Chol) and 3-(4,5-dimethylthiazol-2-yl)-2,5-diphenyltetrazolium bromide (MTT) were obtained from Sigma-Aldrich (St. Louis, MS, USA). 1,2-Distearoyl-sn-glycero-3-phosphoethanolamine-*N*-folate poly-(ethylene glycol)_2000_ (DSPE-PEG_2000_-FA), dioleoylphosphatidylethanolamine (DOPE) and egg l-α-phosphatidylcholine (ePC) were purchased from Avanti Polar Lipids (Alabaster, AL, USA). Branched poly(ethyleneimine) (PEI, *M*_W_ 25 kDa) was purchased from Sigma-Aldrich (USA). The plasmid pCMV-EGFP (pEGFP-N1) carrying enhanced green fluorescent protein (EGFP) under cytomegalovirus (CMV) promoter was propagated in *Escherichia coli* and purified by Endo Free Plasmid Maxi Kit (Qiagen, Hilden, Germany). Hyaluronic acid (HA, 300 kDa) was provided by Shandong Freda Biochem Co., Ltd. (Jinan, China). Agarose was purchased from Biowest (Nuaille, France). DNase I enzyme and goldview was purchased from Solarbio (Shanghai, China). Lipofectamine™ 2000 was purchased from Invitrogen by Life Technologies (Carlsbad, CA, USA). All other reagents were of commercial special grade and used without further purification.

### 3.2. Cell Lines and Cell Culture

Murine melanoma cell line B16 and human hepatoma HepG2 cells were purchased from the Chinese Academy of Sciences (Shanghai, China) and cultured in RPMI-1640 media at 37 °C under 5% CO_2_. All the media were supplemented with 10% (*v*/*v*) fetal bovine serum from Sijiqing Co., Ltd. (Hangzhou, China), streptomycin at 100 μg/mL and penicillin at 100 U/mL.

### 3.3. Preparation of PEI/DNA Complexes

Stock PEI solution (1000 μg/mL) and DNA solution (100 μg/mL) were prepared before each experiment and stocked in hydroxyethyl piperazine ethanesulfonic acid (HEPES)-buffered saline (HBS, pH 7.4). The PEI/DNA complex was prepared at various molar ratios of PEI nitrogen (N) to DNA phosphate (P) up to N/P = 12. Specifically, the PEI/DNA complexes were prepared by slowly mixing the solution of DNA in the PEI solution at N/P ratios of 0.5/1, 1/1, 2/1, 4/1, 6/1, 8/1, 10/1 and 12/1, respectively. Samples were continuously stirred during addition and equilibrated at room temperature for 20 min before measurement. Complexes were freshly prepared before each individual measurement. A gel retardation assay was used to determine the stability of the PEI/DNA complexes. Each well of the gels was loaded with PEI/DNA complexes containing 1 μg of plasmid DNA while the naked DNA served as control. After loading the complexes onto the agarose gel, electrophoresis was carried out in constant voltage mode at 90 V for 20 min.

### 3.4. Preparation of Paclitaxel and PEI-DNA Co-Loaded Liposomes (PPD)

Liposomes were prepared using a slight modification of the previously described method for preparing multi-lamellar vesicles [26]. To prepare empty liposomes, ePC, DOPE and were dissolved in chloroform at a mass ratio of 7:4:2. While PTX-loaded liposomes were being prepared, PTX-chloroform stock solution (1 mg/mL) was combined with the mixed lipids at a mass ratio of 7:4:2:0.8. (ePC:DOPE:Chol:PTX). The mixture was evaporated to dryness in a round-bottomed flask using a rotary evaporator at room temperature. The resulting thin lipid film was dried using nitrogen for an additional 10 min to evaporate any dehydrated chloroform. To prepare PTX/PEI-DNA co-loaded liposomes, 2 mL of PEI/DNA complexes prepared in Section 3.2 was slowly added to the round-bottomed flask and then gently vibrated to facilitate hydration of the thin lipid film. The resulting multi-lamellar liposomes were then sonicated using a Sonic Dismembrator (Model 500; Thermo Fisher Scientific, Pittsburgh, PA, USA) for 3 min at room temperature to form homogenized cationic PPD.

### 3.5. Preparation of FA Modified Paclitaxel and DNA Loaded Liposomes (FA/PPD)

To prepare FA modified PPD, the covalent lipid DSPE-PEG-FA was added to the lipid mixture (5%, *w*/*w*), making the final mass ratio of ePC:DOPE:Chol:DSPE-PEG-FA 7:4:2:0.65. After that, the FA/PPD was also prepared by the modified film dispersion method and details as shown in 3.4.

### 3.6. Preparation of HA-Coated Targeting Liposomes (HA/FA/PPD)

The HA coated FA/PPD was prepared by electrostatic attraction. Briefly, 1 mL of FA/PPD dispersion was slowly added under vigorous stirring to HA solution (0.5 mg/mL, pH 6.0) at mass ratio of 0:1, 0.5:1, 1:1, 2:1, 3:1 and 4:1 (HA: FA/PPD, *w*/*w*). After incubation for 20 min at room temperature, the HA-coated FA/PPD (HA/FA/PPD) was finally obtained.

### 3.7. Characterization of FA/PPD and HA/FA/PPD

The morphology of FA/PPD and HA/FA/PPD was investigated by transmission electron microscopy (TEM) (JEM-1200EX, JEOL Ltd., Tokyo, Japan). For TEM studies, the FA/PPD and HA/FA/PPD dispersion were dropped on the surface of copper grid, and then 2% aqueous solution of sodium phosphotungstate was added for negative staining. After air-drying, the copper grid was placed to TEM and investigated. The particle size and zeta potential of FA/PPD and HA/FA/PPD were measured by laser light scattering using Zetasizer 3000SH (Malvern Instruments Ltd., Malvern, UK). All measurements were carried out in triplicate.

### 3.8. Stability of HA/FA/PPD in the Presence of Plasma

To estimate the stability of HA/FA/PPD under physiologically relevant conditions, the turbidity change of HA/FA/PPD suspension in the presence of plasma was evaluated. Specifically, a freshly prepared HA/FA/PPD solution (200 µL) was added to 1 mL of 50% plasma at pH 7.4 and the mixed solution was incubated at 37 °C with mild stirring. The turbidity of the mixed solution was measured by an ultraviolet-visible spectrophotometer (UV-2102PC; Unico Ltd., Dayton, NJ, USA) at 450 nm after 0.08, 0.17, 0.25, 0.33, 0.5, 1, 2, 4, 5, 12, 16, 20 and 24 h of incubation, respectively. FA/PPD with same treatment served as control.

### 3.9. DNase I Protection Assay

To test whether HA/FA/PPD and FA/PPD could protect loaded DNA from nuclease degradation, DNase I-mediated degradation was evaluated using agarose gel electrophoresis. In brief, 20 µL of HA/FA/PPD and FA/PPD containing 1 µg of DNA was incubated with DNase I (0.2 U/µg DNA) in DNase I buffer. The suspensions were incubated in a shaking water bath at 37 °C and 100 rpm for 0.5, 1, 2, 3 and 4 h, respectively. After incubation, ethylenediaminetetraacetic acid solution (0.5 M, pH 8.0) was added to terminate the enzymatic degradation reaction. To separate DNA from HA/FA/PPD and FA/PPD, TE buffer containing 1% heparin was added to the suspensions. The suspensions were then placed in a 37 °C shaking water bath at 100 rpm for another 2 h. After that, the fluorescent intensity of bands corresponding to DNA and their electrophoretic mobility were analyzed by gel electrophoresis. Naked DNA (1 µg) incubated with DNase I for 30 min served as control.

### 3.10. In Vitro Release Studies

The *in vitro* release of PTX from HA/FA/PPD and FA/PPD was evaluated by dynamic dialysis method [27]. Briefly, HA/FA/PPD and FA/PPD suspensions were diluted with de-ionized water to final PTX concentration of 100 µg/mL and placed into a pre-swelled dialysis bag with 8–12 kDa molecular weight cutoff. The dialysis bag was incubated in 15 mL release media (PBS containing 0.5% of Tween-80) at 37 ± 0.5 °C with stirring at 100 rpm. At predetermined time intervals, 1 mL solution was taken out and 15 mL fresh media was filled to replace the remaining release media. The amount of PTX in samples was then determined by high performance liquid chromatography (HPLC) method.

### 3.11. In Vitro Cytotoxicity Studies

Murine melanoma cell line B16 and human hepatoma HepG2 cells were selected to investigate the *in vitro* cytotoxicity study. The *in vitro* cytotoxicity of HA/FA/PPD and FA/PPD on B16 and HepG2 cells were investigated by MTT assay. All concentrations were expressed in PTX equivalents. In brief, cells were seeded into a 96-well plate at a density of 4000 cells/well. After 48 h incubation with a series of doses of Taxol^®^, HA/FA/PPD and FA/PPD (final PTX concentration were 0.1, 0.5, 1, 5 and 10 μg/mL, respectively), 20 μL of MTT solution in PBS (5 mg/mL) was added to each well. After another 4 h, the incubation media was removed and 200 μL of DMSO was subsequently added to each well to dissolve formazan crystals, and measured at 570 nm. Moreover, to evaluate the safety of the prepared nanovector, the FA/PD (PTX free FA/PPD) and the HA/FA/PD (PTX free HA/FA/PPD) were prepared with the abovementioned method and the cytotoxicity at different DNA concentrations (1, 2, 3, 4, and 5 μg/mL) were investigated by abovementioned method. All experiments were repeated independently thrice and the relative cell viability (%) compared to control cells was calculated.

### 3.12. In Vitro Transfection Study

The transfection activity of HA/FA/PPD and FA/PPD on B16 cells was evaluated using plasmid pCMV-EGFP (pEGFP-N1) carrying enhanced green fluorescent protein (EGFP) as reporter gene. Briefly, B16 cells were seeded in six-well culture plates at a density of 3 × 10^5^ cells per well and incubated overnight at 37 °C, 5% CO_2_. Before experiments, the growth media was removed and the cells were washed twice with pH 7.4, 0.01 M phosphate-buffered saline (PBS). Serum free cell culture media or 10% serum cell culture media containing HA/FA/PPD or FA/PPD were subsequently added. HA/FA/PPD and FA/PPD under different culture media were added at 2 μg of pDNA per well, respectively. After incubation for 4 h at 37 °C, the transfection media containing tested HA/FA/PPD or FA/PPD were removed and washed twice with PBS. Fresh RPM-1640 culture media containing 10% serum was added to each well and the cells were incubated for 48 h at 37 °C. Lipofectamine™ 2000/pDNA complexes containing equivalent pDNA were served as control. Fluorescence in cells was observed using an inverted fluorescence microscope (Olympus Corporation, Tokyo, Japan). After that, all cells were harvested and washed in PBS for three times, and the fluorescence intensity was quantitatively determined by a FACSCalibur™ fow cytometer (BD Biosciences, San Jose, CA, USA) by counting 10,000 events. Only the viable cells were gated for fluorescence analysis.

### 3.13. In Vitro Targeting Cellular Uptake Studies

To investigate whether the dual targeting HA/FA/PPD showed advantages in cellular uptake, Rho-PTX labeled HA/FA/PPD, FA/PPD and PPD (10%, *w*/*w*) were prepared, respectively, and B16 cell was chosen as the CD44-positive and FA-positive cell line [20,21]. The cellular uptake of dual targeting HA/FA/PPD was quantified by flow cytometry. Briefly, B16 cells were seeded in 12-well culture plates at a density of 1 × 10^5^ cells per well for overnight. After the cells reached 80% confluence, fresh media containing 2 μg/mL of Rho-PTX labeled HA/FA/PPD, FA/PPD and PPD were added, respectively, and incubated for 0.5 and 2 h at 37 °C, 5% CO_2_ incubator. Then the media containing tested samples were removed and cells were harvested. The fluorescence intensity of the cells with different treatment was measured by flow cytometer (BD Biosciences). For each sample, 10,000 events were collected and the percentage of positive events was calculated as the events within the gate divided by the total number of events, excluding cell debris.

### 3.14. Co-Delivery of PTX and pDNA into Tumor Cells

To study whether pDNA and PTX could be co-delivered into the same cancer cell to achieve the potential synergistic cancer treatment, Rho-PTX labeled HA/FA/PPD, FA/PPD were prepared and HepG2 was chosen as the CD44-positive and FA-negative cell line [22,23]. Fluorescence microscope and a two-color flow cytometry were used to determine the co-delivery of PTX and pDNA. Briefly, HepG2 cells were seeded in six-well culture plates at a density of 3 × 10^5^ cells per well and incubated overnight at 37 °C, 5% CO_2_. Before experiments, the growth media was removed and the cells were washed twice with PBS. Serum free culture media containing HA/FA/PPD or FA/PPD were subsequently added. HA/FA/PPD and FA/PPD were added at 2 μg of pDNA and 2 μg/mL of Rho-PTX per well, respectively. After incubation for 4 h at 37 °C, the media containing tested HA/FA/PPD or FA/PPD were removed and washed twice with PBS buffer. Fresh RPM-1640 culture media containing 10% serum was added to each well and the cells were incubated for 48 h at 37 °C. Samples were observed under an inverted fluorescence microscope (Olympus Corporation, Tokyo, Japan) and quantitatively determined by a FACSCalibur™ flow cytometer (BD Biosciences).

### 3.15. Statistical Analysis

All studies were repeated a minimum of three times and measured at least in triplicate. Results were reported as mean ± SD (standard deviation). Statistical significance was analyzed using Student’s *t*-test. Differences between experimental groups were considered significant when *p* < 0.05.

## 4. Conclusions

In the present study, HA- and FA-modified dual targeting biomimetic liposomes were prepared to co-deliver PTX and DNA (HA/FA/PPD). The outermost anionic HA layer was shown to significantly reduce the toxicity of the cationic FA/PPD and enhance the stability of the nanovectors. More importantly, compared to FA/PPD and PPD, the dual targeting properties of HA/FA/PPD could significantly improve transfection efficacy and enhance cellular uptake. Moreover, HA/FA/PPD could effectively co-deliver PTX and DNA into the same tumor cells, offering great potential for combination cancer treatment. In conclusion, the obtained dual targeting biomimetic liposomes showed great superiority in targeting to tumor cells, enhancing cellular uptake and transfection efficiency *in vitro*, and are worthy of further study *in vivo* in the near future.
